# PD-1^hi^TIM-3^+^ T cells associate with and predict leukemia relapse in AML patients post allogeneic stem cell transplantation

**DOI:** 10.1038/bcj.2015.58

**Published:** 2015-07-31

**Authors:** Y Kong, J Zhang, D F Claxton, W C Ehmann, W B Rybka, L Zhu, H Zeng, T D Schell, H Zheng

**Affiliations:** 1 Penn State Hershey Cancer Institute, Penn State University College of Medicine, Hershey, PA, USA; 2 Institute of Infectious Diseases, Beijing Ditan Hospital, Capital Medical University, Beijing Key Laboratory of Emerging Infectious Diseases, Beijing, China; 3 Department of Microbiology and Immunology, Penn State University College of Medicine, Hershey, PA, USA

## Abstract

Prognosis of leukemia relapse post allogeneic stem cell transplantation (alloSCT) is poor and effective new treatments are urgently needed. T cells are pivotal in eradicating leukemia through a graft versus leukemia (GVL) effect and leukemia relapse is considered a failure of GVL. T-cell exhaustion is a state of T-cell dysfunction mediated by inhibitory molecules including programmed cell death protein 1 (PD-1) and T-cell immunoglobulin domain and mucin domain 3 (TIM-3). To evaluate whether T-cell exhaustion and inhibitory pathways are involved in leukemia relapse post alloSCT, we performed phenotypic and functional studies on T cells from peripheral blood of acute myeloid leukemia patients receiving alloSCT. Here we report that PD-1^hi^TIM-3^+^ cells are strongly associated with leukemia relapse post transplantation. Consistent with exhaustion, PD-1^hi^TIM-3^+^ T cells are functionally deficient manifested by reduced production of interleukin 2 (IL-2), tumor necrosis factor-α (TNF-α) and interferon-γ (IFN-γ). In addition, these cells demonstrate a phenotype consistent with exhausted antigen-experienced T cells by losing T_N_ and T_EMRA_ subsets. Importantly, increase of PD-1^hi^TIM-3^+^ cells occurs before clinical diagnosis of leukemia relapse, suggesting their predictive value. Results of our study provide an early diagnostic approach and a therapeutic target for leukemia relapse post transplantation.

## Introduction

Leukemia relapse remains the top cause of death post allogeneic hematopoietic stem cell transplantation (alloSCT) in patients with acute myeloid leukemia (AML).^1^ Once leukemia relapse occurs, the prognosis is generally poor with the overall 5-year survival of only 5% and medium time to death of 3–4 months.^2, 3^ Treatment options in this population are extremely limited. General management includes withdrawal of immune suppressors, reinduction chemotherapy, donor lymphocyte infusion and second transplantation.^4, 5, 6, 7, 8, 9, 10, 11^ None of these approaches are very effective. Instead, they all carry some degree of risk such as graft versus host disease (GVHD), severe infections and multiorgan failure. The complications are often severe and life threatening. Currently, there is no ‘standard care' for patients with AML relapse post alloSCT and clinical practice is largely per physician's choice. Clearly, novel effective leukemia therapeutics is urgently needed.

Eradication of leukemia in alloSCT largely relies on graft versus leukemia (GVL) mediated by donor T cells that are also involved in GVHD.^12, 13^ Leukemia relapse is considered a failure of GVL. Much effort has been placed on enhancing the GVL effect, although little progress has been achieved in the past four decades. Inhibitory mechanisms play pivotal roles in tumor evasion from immune attack. Targeting inhibitory mechanisms by blocking negative pathways, the so-called immune checkpoints, have recently been proved safe and effective in treating several types of solid tumors.^14, 15, 16, 17^ T-cell exhaustion is a unique immune inhibitory mechanism. It is a state of T-cell dysfunction that develops in response to persistent antigen stimulation.^18^ Exhausted T cells lose their capacity for production of cytokines such as interleukin 2 (IL-2), tumor necrosis factor-α (TNF-α) and interferon-γ (IFN-γ), as well as the ability to proliferate and perform cytotoxic killing.^19, 20, 21, 22^ Eventually they undergo apoptosis and deletion.^19, 23^ Inhibitory pathways including programmed cell death protein 1 (PD-1), T-cell immunoglobulin domain and mucin domain 3 (TIM-3), 2B4, CD160, B- and T-lymphocyte attenuator (BTLA) and lymphocyte-activation gene 3 (LAG-3) are tightly associated with T-cell exhaustion.^18^ They are not only significant markers for the status of exhaustion, but are also key mediators causing the hyporesponsiveness of exhausted T cells.

T-cell exhaustion was first demonstrated in chronic viral infections and more recently in the setting of cancer.^23, 24, 25, 26, 27, 28, 29, 30, 31^ In alloSCT, alloantigen-reactive T cells are classically thought to be highly reactive, but this setting also provides persistent antigen that is ideal for induction of T- cell exhaustion. We hypothesize that T-cell exhaustion contributes to GVL failure and leukemia relapse post alloSCT, therefore targeting key mediators of T-cell exhaustion to regain T-cell activity, and the GVL effect is a promising leukemia therapeutic. In this study, we performed phenotypic and functional studies on T cells from peripheral blood of AML patients receiving alloSCT. Cells expressing negative receptors involved in T-cell exhaustion were evaluated. We report that PD-1^hi^TIM-3^+^ cells are strongly associated with leukemia relapse post transplantation. Consistent with exhaustion, PD-1^hi^TIM-3^+^ T cells produced low intracellular IL-2, TNF-α and IFN-γ. Importantly, PD-1^hi^TIM-3^+^ T cells have predictive value for leukemia relapse post alloSCT.

## Materials and methods

### Patients

Peripheral blood samples were collected from AML patients from the tissue bank maintained by the Penn State Hershey Cancer Institute of Penn State University College of Medicine (Hershey, PA, USA). The study was approved by the institutional review board of Penn State University College of Medicine. Full informed consent was obtained from all patients. Samples from 11 AML patients who received alloSCT from 2013 to 2015 were selected, 5 of whom had leukemia relapse at 2–6 months post transplantation; the other 6 patients remained in remission at the time of blood collection (3–6 months). All patients had a diagnosis of AML per World Health Organization classification. Age and gender were evenly distributed in relapse vs remission patients. Most patients received 8/8 human leukocyte antigen-matched transplantations from filgrastim-mobilized peripheral blood stem cells. One patient received 7/8 matched donor cells. One patient received bone marrow-derived stem cells. Seven patients (3 from relapse and 4 from remission group) underwent ablative conditioning, and the other 4 patients (two from each group) received nonablative conditioning regimens because of age or comorbidity. The clinical characteristics of these patients are summarized in Table 1.

### Isolation of PBMCs

Peripheral blood samples were collected from patients with AML at indicated time points post alloSCT. Blood was diluted 1:1 with phosphate-buffered saline before separation of peripheral blood mononuclear cells (PBMCs) with Ficoll-Paque (Amersham Pharmacia Biotech, Stockholm, Sweden) density gradient centrifugation. Cells were cryopreserved in fetal bovine serum (GIBCO, Grand Island, NY, USA) supplemented with 10% dimethyl sulfoxide and stored in liquid nitrogen.

### Immunofluorescence staining and flow cytometric analysis

For surface staining, PBMCs from liquid nitrogen were thawed and washed twice in phosphate buffered saline containing 1% fetal bovine serum (staining buffer). Cells were incubated with directly conjugated monoclonal antibodies for 30 min at 4 °C. The cells were then washed and resuspended in staining buffer before flow cytometric analysis. The monoclonal antibodies used were anti-human CD3-PerCp-Cy5.5 or CD3-BV421, CD4-FITC or CD4-V500, CD8-APC-H7, CD45RA-PE-Cy7, CCR7-PerCp-Cy5.5 (BD Biosciences, San Diego, CA, USA), PD-1-PE, TIM-3-FITC, 2B4-APC, BTLA-BV421, CD160-PE-Cy7 (BioLegend, San Diego, CA, USA) and LAG-3-AF700 (R&D Systems, Minneapolis, MN, USA) antibodies, and corresponding isotype controls. Data acquisition was performed on a LSR Fortessa flow cytometer (BD Biosciences) and data analysis was performed using FlowJo Software (Tree Star, Ashland, OR, USA).

### *In vitro* stimulation and intracellular cytokine staining

PBMCs were cultured in RPMI-1640 medium (GIBCO) containing 10% fetal bovine serum and stimulated with anti-CD3/CD28 (2 and 5 μg/ml) antibodies or phorbol 12-myristate 13-acetate (PMA)/ionomycin (50 ng/ml and 1 μg/ml, respectively), plus Golgiplug (BD Pharmingen, San Diego, CA, USA) for 5 h. The cells were then surface stained with CD4-FITC, CD8-APC-H7, PD-1-PE and TIM-3-PE-Cy7, and intracellularly stained with IFN-γ-PE-CF594, TNF-α-PerCp-Cy5.5 or IL-2-PerCp-Cy5.5 (BD Pharmingen) antibodies. A violet amine reactive dye (Invitrogen, Grand Island, NY, USA) was used to assess cell viability.

### Statistical analysis

Data are expressed as the mean±s.d. GraphPad5 (GraphPad Software, La Jolla, CA, USA) was used for statistical calculations. Comparisons between groups were analyzed using independent-sample *t-*tests. For all analyses, *P-*values <0.05 were considered statistically significant.

## Results

### T cells from patients with AML relapse post alloSCT have elevated expression of PD-1 and TIM-3

**Figure 1:**
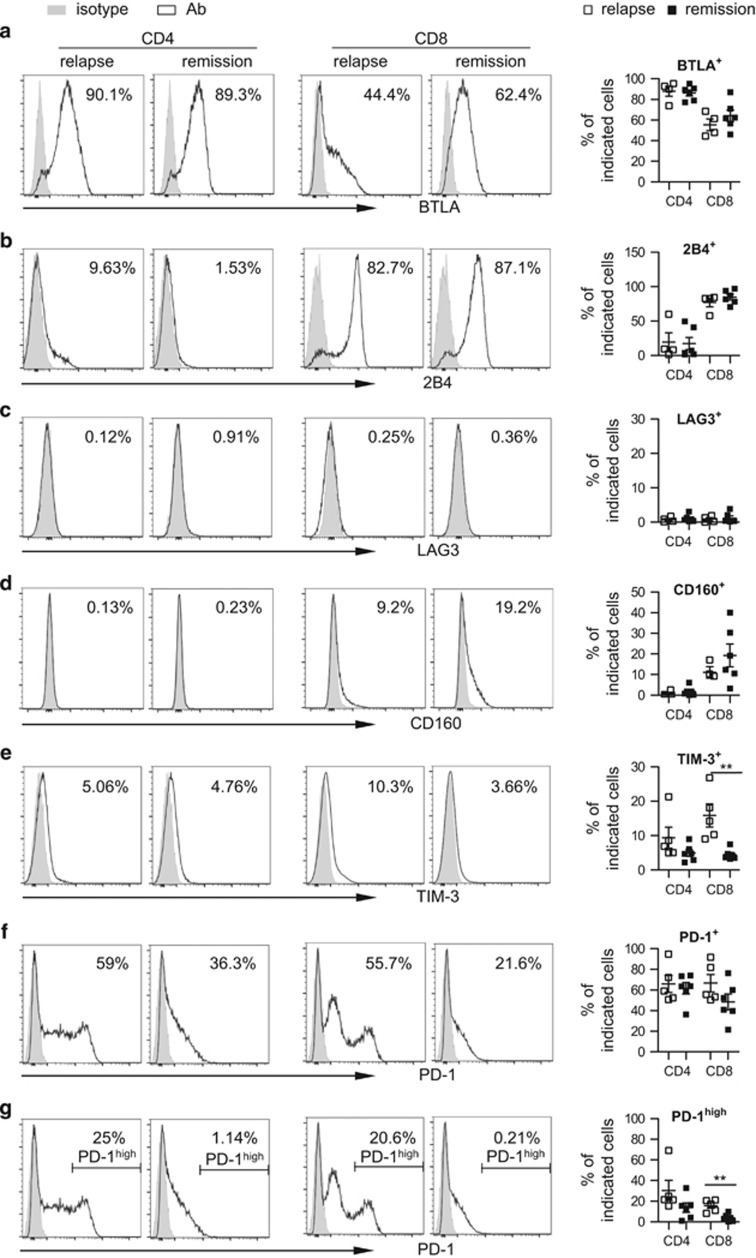
Expression of PD-1 and TIM3 is enhanced on T cells from patients with leukemia relapse post transplant. Flow cytometry analysis of surface expression of BTLA, 2B4, LAG3, CD160, TIM-3 and PD-1 was performed on PBMCs collected from AML patients post alloSCT. (**a–g**) Histogram displays the expression of above receptors on T cells from representative patients of relapse vs remission. CD4^+^ or CD8^+^ cells were gated. Percentage of cells expressing each receptor is shown. Panels on right are plots of expression of each receptor on CD4^+^ or CD8^+^ T cells from patients with relapse (*n*=4–5) vs remission (*n*=6). Each square represents data from one patient. **P<0.01.

It has been reported that several receptors including BTLA, 2B4, LAG-3, CD160, TIM-3 and PD-1 are upregulated on the T-cell surface to mediate T-cell exhaustion in a variety of tumors.^18^ To determine whether these exhaustion markers associate with AML relapse post transplantation, we performed flow cytometric analysis of these molecules using PBMCs from 11 patients post alloSCT, among whom 5 had leukemia relapse whereas the other 6 remained in remission at the time of sample collection. Table 1 outlines the patients' clinical characteristics. Expressions of BTLA, 2B4, LAG-3 and CD160 were comparable between relapse and remission patients (Figures 1a–d), whereas the percentage of TIM-3^+^ cells among CD8^+^ T cells was significantly higher in patients who had leukemia relapse (*P*=0.0051; Figure 1e). CD4^+^ T cells showed a similar trend but did not achieve statistical significance (*P*=0.1684; Figure 1e). Importantly, although the percentage of overall PD-1^+^ cells showed no difference (Figure 1f), we consistently detected an increase in the proportion of cells expressing high levels of PD-1 (PD-1^hi^). Consistent with TIM-3 data, PD-1^hi^ CD8^+^ T cells were more predominant in relapse patients compared with remission patients (*P*=0.0025; Figure 1g). CD4^+^ T cells showed the same trend, although not statistically significant (*P*=0.1359; Figure 1g). These data suggest a role for TIM-3 and PD-1 in leukemia relapse post alloSCT.

### PD-1^hi^TIM-3^+^ T cells are associated with leukemia relapse in AML patients who received alloSCT

**Figure 2:**
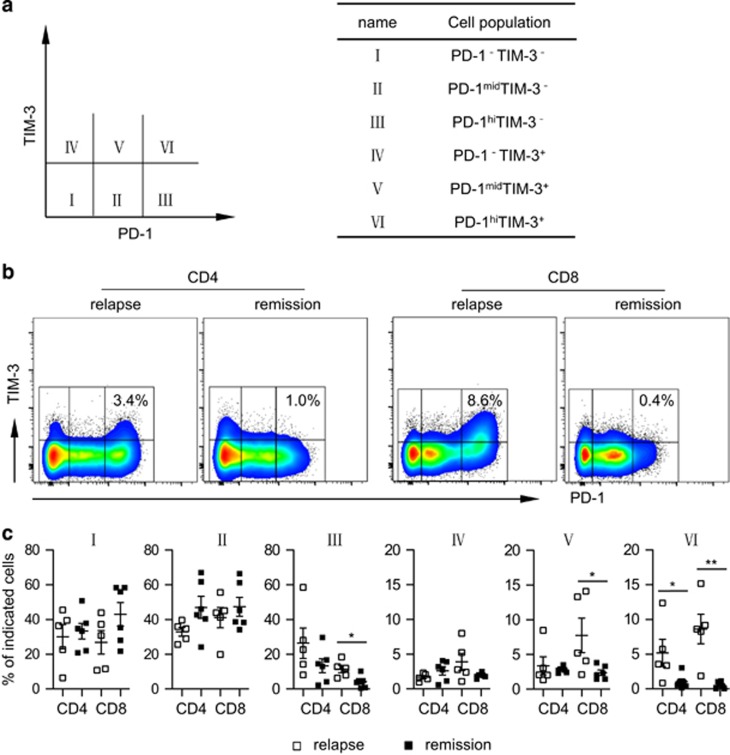
PD-1^hi^TIM3^+^ cells associate with leukemia relapse post alloSCT. PBMCs from patients with leukemia relapse vs remission were tested for PD-1 and TIM3 expression on CD4^+^ and CD8^+^ T cells by flow cytometry. (**a**) Based on levels of PD-1 and TIM3 expression, cells are divided into six fractions. Shown is the schema of each fraction. (**b**) Representative flow data from one relapse (patient 09) and one remission (patient 02). Percentage of PD-1^hi^TIM-3^+^ among CD4^+^ or CD8^+^ cells is shown. (**c**) Plot of percentage of each fraction among CD4^+^ or CD8^+^ cells in patient with relapse (*n*=5) vs remission (*n*=6). Each square represents data of an individual patient. *P<0.05, **P<0.01.

We further dissected the association between expression of PD-1 and TIM-3 with leukemia relapse. Based on the level of PD-1 vs TIM-3, both CD4^+^ and CD8^+^ T cells can be divided into six subpopulations as shown in Figure 2a. Critically, we detected significantly higher frequencies of PD-1^hi^TIM-3^+^ (fraction VI) cells in samples from patients with leukemia relapse compared with that of patients who remained in remission. The median frequencies of CD8^+^ T cells expressing PD-1^hi^ TIM-3^+^ were 8.6%, compared with 0.5% in patients maintaining remission (*P*=0.0022; Figures 2b and c). This phenomenon occurred in CD4^+^ T cells as well (*P*=0.0496; Figures 2b and c). Fraction III (PD-1^hi^TIM-3^−^) and fraction V (PD-1^med^TIM-3^+^) showed a pattern similar to fraction VI, but only CD8^+^ T cells exhibited statistical significances (*P*=0.0155 and 0.0429 for fraction III and V, respectively; Figures 2b and c). This is an important finding suggesting that PD-1^hi^TIM-3^+^ T cells are associated with leukemia relapse post alloSCT.

### PD-1^hi^TIM-3^+^ T cells are dysfunctional as manifested by reduced cytokine production

**Figure 3:**
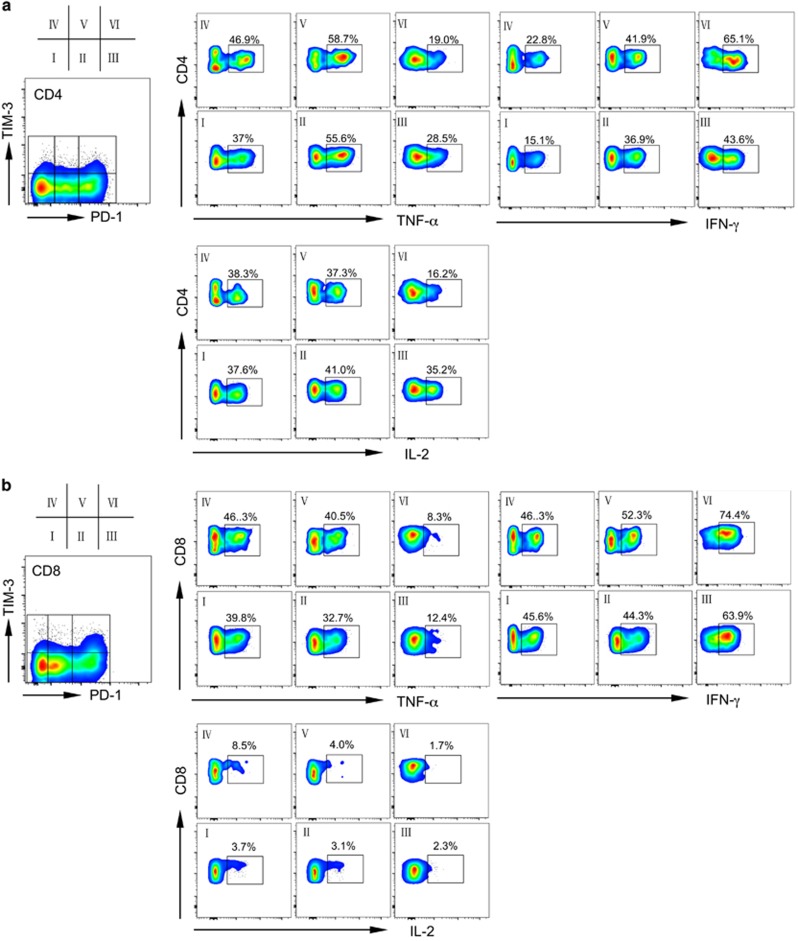
PD-1^hi^TIM3^+^ cells produce less TNF-a and IL-2 upon PMA/ionomycin stimulation. Production of TNF-α, IFN-γ and IL-2 upon *in vitro* PMA/ionomycin stimulation. Shown is cytokine release from CD4^+^ (**a**) or CD8^+^ (**b**) T cells gated on each fraction of cells based on PD-1 and TIM-3 expression. Representative plots from a single relapse patient (09) are shown.

**Figure 4:**
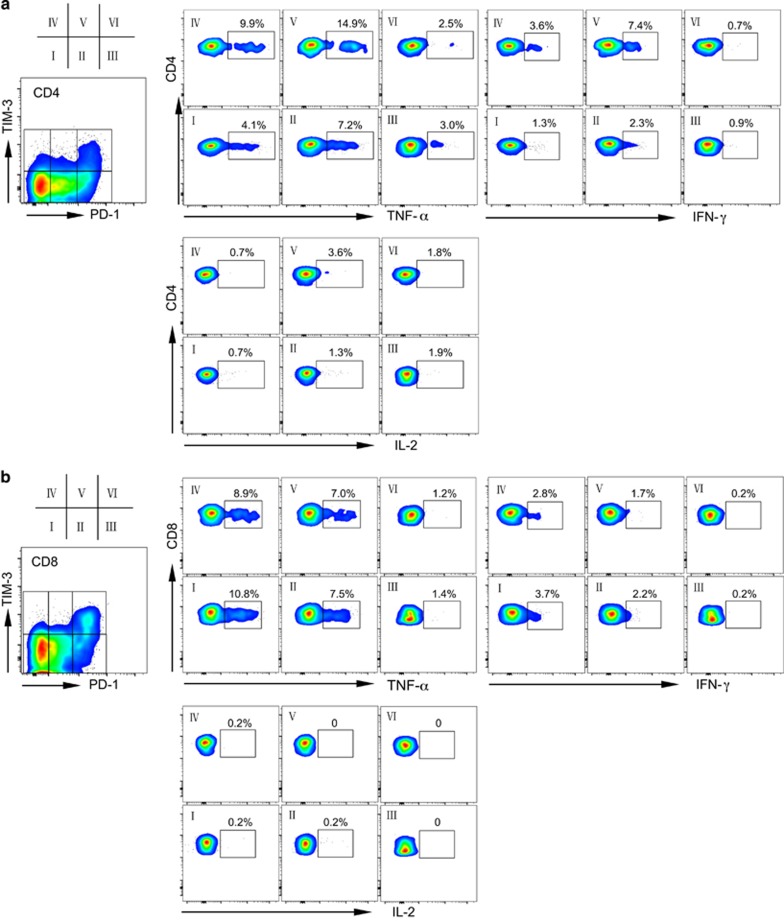
PD-1^hi^TIM3^+^ cells produce less cytokines in response to anti-CD3/anti-CD28 stimulation. Production of TNF-α, IFN-γ and IL-2 upon *in vitro* anti-CD3/anti-CD28 stimulation. Shown is cytokine release from CD4^+^ (**a**) or CD8^+^ (**b**) T cells gated on each fraction of cells based on PD-1 and TIM-3 expression. Representative plots from a single relapse patient (09) are shown.

To evaluate the functional status of PD-1^hi^TIM-3^+^ T cells, we performed functional assays to test cytokine release by T cells derived from AML patients with leukemia relapse post transplantation. T cells were stimulated *in vitro* before flow cytometry analysis for intracellular IL-2, TNF-α and IFN-γ production. The cells were costained with PD-1 and TIM-3, allowing us to dissect the function of each T-cell fraction based on PD-1 and TIM-3 expression. We first used PMA/ionomycin as the *in vitro* stimulator for T cells. Figure 3 shows the cytokine release of T cells from a patient with AML relapse post transplantation (patient 09). The PD-1^hi^TIM-3^+^ population in CD4^+^ T cells produced much lower TNF-α and IL-2 compared with that from fractions I, II, IV and V (19.0% vs 37–58.7% of TNF-α release, and 16.2% vs 37.3–41% of IL-2 release; Figure 3a). Fraction III showed moderate loss of TNF-α (28.5%) and IL-2 (35.2%). A similar pattern was observed for CD8^+^ T cells, in that PD-1^hi^TIM-3^+^CD8^+^ T cells produced extremely low TNF-α and IL-2 (Figure 3b). Interestingly, in both CD4^+^ and CD8^+^ T cells, the PD-1^hi^TIM-3^+^ population had increased production of IFN-γ (Figures 3a and b). PMA/ionomycin stimulates T-cell activation through a strong but non-T-cell receptor (TCR)-dependent pathway.^32^ To dissect the T-cell functional status by mimicking the physiological signal, we performed a separate study of cytokine release using anti-CD3 and anti-CD28 as *in vitro* stimulators, in which the activation of T cells is TCR dependent. As expected, the overall levels of cytokine release were reduced following CD3/CD28 stimulation compared with that obtained with PMA/ionomycin stimulation (Figure 4). Consistent with results obtained using PMA/ionomycin stimulation, PD-1^hi^TIM-3^+^ CD4^+^ T cells had much lower intracellular TNF-α and IL-2. Importantly, IFN-γ production was also decreased in this population (Figure 4a). CD8^+^ T cells followed the same pattern (Figure 4b) in that PD-1^hi^TIM-3^+^CD8^+^ T cells produced low TNF-α and IFN-γ. As expected, CD8^+^ T cells did not produce much IL-2. These results indicate that the PD-1^hi^TIM-3^+^ T cells that developed in leukemia relapse are dysfunctional, consistent with development of exhaustion.

### PD-1^hi^TIM-3^+^ cells are phenotypically antigen-experienced exhausted T cells

**Figure 5:**
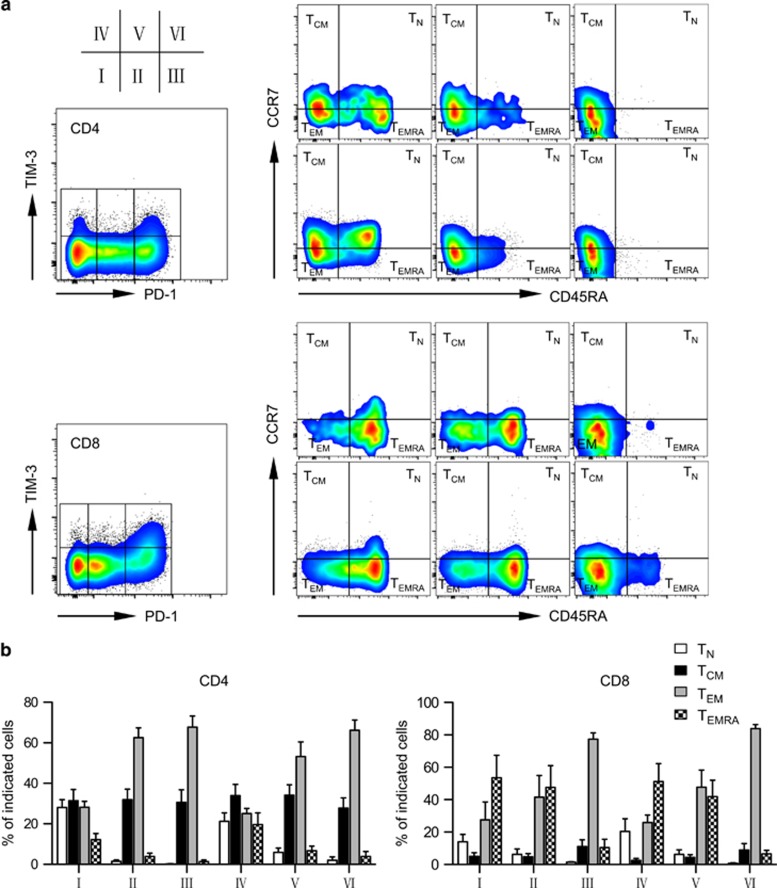
PD-1^hi^TIM3^+^ cells lose T_EMRA_ in patients with leukemia relapse post transplant. Distribution of T_N_, T_CM_, T_EM_ and T_EMRA_ in T cells gated on each fraction of cells based on PD-1 and TIM-3 expression from relapse patients. (**a**) Representative dot plots from one relapse patient (09). (**b**) Summary data for five relapse patients (08, 09, 10, 11 and 12).

Based on the expression of CD45RA and CCR7, T cells are generally divided into four subsets: naive T cells (T_N_, CCR7^+^CD45RA^+^), central memory T cells (T_CM_, CCR7^+^CD45RA^-^), effector memory T cells (T_EM_, CCR7^-^CD45RA^−^) and terminally differentiated effector cells (T_EMRA_, CCR7^-^CD45RA^+^).^33, 34^ By multichannel flow cytometry, we examined the distribution of all these four subsets among PD-1^hi^TIM-3^+^ T cells (fraction VI) as well as other T-cell fractions (fractions I–V) based on PD-1 and TIM-3 expression. In contrast to other fractions that mostly consist of all four T-cell subsets, PD-1^hi^TIM-3^+^ T cells are clearly antigen experienced as they had entirely lost the T_N_ subset. The majority of PD-1^hi^TIM-3^+^ cells are T_CM_ or T_EM_. Interestingly, T_EMRA_ also are significantly decreased in this cell fraction. This occurred in both CD4^+^ and CD8^+^ T cells from leukemia relapse patients (Figure 5). Similar patterns of expression were observed in patients maintained in remission (Supplementary Figure 1). Of note, fraction III (PD-1^hi^TIM-3^−^) cells are phenotypically shifted toward the stage of PD-1^hi^TIM-3^+^ cells by loss of the T_N_ subset and minimal accumulation of the T_EMRA_ subset. We performed functional analysis to evaluate cytokine release of each T-cell subset using cells from one relapse patient (patient 11). T_EMRA_ play a dominant role in producing TNF-α and IFN-γ in both CD4^+^ and CD8^+^ T cells (Supplementary Figure 2). Thus, loss of T_EMRA_ might contribute to the functional defect of PD-1^hi^TIM-3^+^ T cells. Taken together, these results support that PD-1^hi^TIM-3^+^ cells are phenotypically antigen-experienced T cells that have lost functional subsets, consistent with a state of T-cell exhaustion.

### PD-1^hi^TIM-3^+^ T cells have predictive value for leukemia relapse post alloSCT

**Figure 6:**
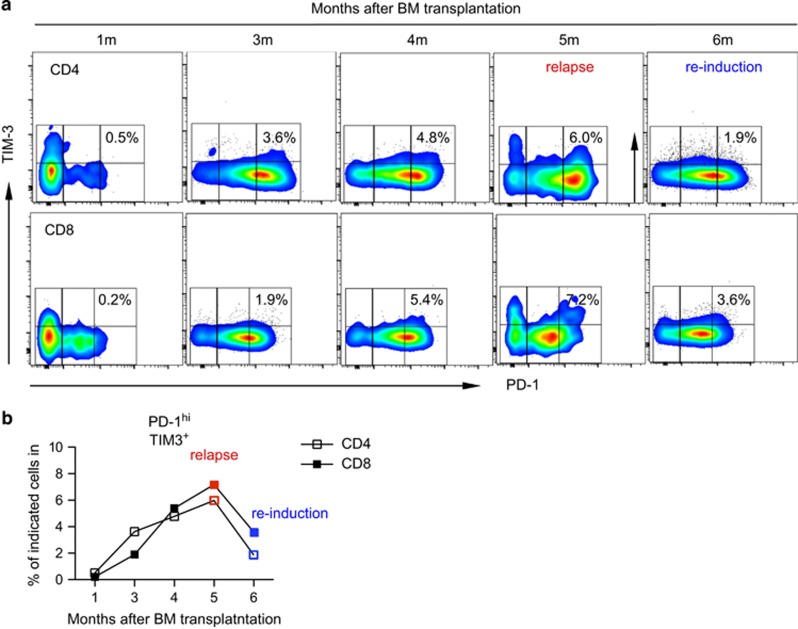
Increase of PD-1^hi^TIM3^+^ cells predict leukemia relapse. PBMCs from a patient (08) who had leukemia relapse 5 month post alloSCT were collected at different time points post transplant. (**a**) Flow cytometry data of expression of PD-1^hi^TIM3^+^ cells among CD4^+^ or CD8^+^ T cells. (**b**) Plot of the percentage of PD-1^hi^TIM-3^+^ cells among CD4^+^ or CD8^+^ T cells kinetically post transplantation.

To determine whether PD-1^hi^TIM-3^+^ T cells predict leukemia relapse in AML patients post alloSCT, we evaluated serial samples at 1, 3, 4, 5 (relapse point) and 6 months (achieved remission after reinduction chemotherapy) post transplantation. Cells were evaluated from patient 08 who received matched unrelated donor transplantation for high-risk AML. She was initially doing well until 5 month after transplantation when thrombocytopenia was noted and bone marrow biopsy confirmed leukemia relapse. She then received reinduction chemotherapy and was able to achieve a second complete remission. PBMCs were analyzed by flow cytometry for expression of PD-1 and TIM-3 on CD4^+^ and CD8^+^ T cells. As shown in Figure 6, the percentage of PD-1^hi^TIM-3^+^ T cells started to increase as early as 3 months post transplantation, which is 2 months before clinical diagnosis of relapse. It increased further at 4 months post transplantation and reached the highest level at 5 months, the time of clinical diagnosis of relapse. This pattern is consistent in both CD4^+^ and CD8^+^ T cells. Importantly the percentage of PD-1^hi^TIM-3^+^ T cells decreased at 6 month post transplantation, at which time this patient regained remission after a course of reinduction chemotherapy. In contrast, the percentage of PD-1^hi^TIM-3^+^ cells remained at low levels in patients maintained in remission post transplantation (Supplementary Figure 3). Thus, elevation of the percentage of PD-1^hi^TIM-3^+^T cells can be considered as a predictive biomarker for leukemia relapse post transplantation.

## Discussion

In this study we evaluated the phenotype and function of peripheral blood T cells collected from AML patients who had received alloSCT to determine the relationship of T-cell exhaustion with leukemia relapse. We have made three important findings. (1) PD-1^hi^TIM-3^+^ cells are strongly associated with leukemia relapse post transplantation. The percentage of this cell fraction among both CD4^+^ and CD8^+^ T cells is significantly higher in patients with leukemia relapse compared with patients in remission. (2) Consistent with exhaustion, PD-1^hi^TIM-3^+^ T cells demonstrate a phenotype of exhausted antigen-experienced cells by losing T_N_ and T_EMRA_ subsets. Importantly, these cells produce low intracellular IL-2, TNF-α and IFN-γ. (3) Increase of PD-1^hi^TIM-3^+^ cells occurs before clinical diagnosis of leukemia relapse, thus offering predictive value for leukemia relapse post alloSCT.

Evidence supporting the role for inhibitory receptors and exhaustion in solid tumors has been well defined. Their involvement in chronic lymphocytic leukemia and chronic myeloid leukemia has also been demonstrated clinically.^27, 31, 35^ However, studies of these inhibitory pathways in AML progression are limited and mostly preclinical. In a mouse model of AML, in which AML was induced by intravenous injection of C1498 (a murine leukemia cell line), elevation of PD-L1 on leukemia cells was observed, and less leukemia progression was achieved in PD-1^−/−^ mice as well as by PD-L1 blockade.^36^ In the same mouse model of AML, coexpression of PD-1 and TIM-3 on exhausted CD8^+^ T cells was demonstrated and blockade of PD-1 and TIM-3 pathways synergistically improved relapse-free survival.^30^ Clinical studies using samples from AML patients are scarce. A recent study of gene expression in myelodysplastic syndrome and AML patients demonstrated upregulation of PD-L1 and PD-L2 in leukemia blast, indicating a role for the PD-1 pathway in AML pathogenesis.^37^ In addition, Norde *et al.*^38^ reported that minor histocompatibility antigen-specific T cells expressed elevated PD-1 in a patient during the accelerating phase of chronic myeloid leukemia and a patient with AML post donor lymphocyte infusion. Importantly, blockade with PD-1 antibody restored the proliferation capacity of these minor histocompatibility antigen-specific T cells. These observations suggest that T-cell exhaustion is involved in AML progression and inhibition of alloreactive T-cell responses. However, direct evidence supporting a clinical role for inhibitory pathways and T-cell exhaustion in AML patients who undergo alloSCT has not been provided. Our study on cohorts of patients with AML relapse vs remission post transplantation demonstrates that the PD-1^hi^TIM-3^+^ cells, a fraction of T cells that are characterized as exhausted both phenotypically and functionally, strongly associate with leukemia relapse in AML patients post transplantation. To our knowledge, this is the first clinical evidence directly linking T-cell exhaustion to leukemia relapse in alloSCT. This provides a strong rationale for novel leukemia therapeutics that target inhibitory molecules to reverse exhaustion and therefore restore T-cell function and enhance GVL activity. In fact, nivolumab and pembrolizumab, both of which are antagonistic antibodies to PD-1, were recently approved by the Food and Drug Administration (FDA) to treat advanced melanoma and non-small-cell lung cancer.^14, 15, 16, 17^ Most recently, an early-phase clinical trial using nivolumab in relapse/refractory Hodgkin's lymphoma reported positive results.^39^ Importantly, adverse effects of these agents are minimal. These results have raised a great deal of enthusiasm in the field of cancer therapy and a large number of clinical studies testing the safety and efficacy of agents targeting PD-1 or other inhibitory pathways in multiple types of tumors are currently ongoing.^40^ Our study provides pivotal information to move this strategy forward to leukemia treatment.

A major concern of targeting inhibitory mechanisms such as PD-1 and TIM-3 in treating leukemia relapse post alloSCT is the potential risk of GVHD. It has been shown in a mouse model that blockade of PD-1/PD-L1 improves GVL, but accelerates GVHD.^41^ However, data from the clinical trials testing PD-1 targeting agents in patients with solid tumors demonstrated only minimal side effects. In particular, autoimmune symptoms such as severe diarrhea, pneumonitis and thyroid dysfunction were much less frequently observed compared with those who received ipilimumab, another FDA-approved agent targeting a negative immune modulator.^42, 43, 44^ Despite the potential for autoimunity, ipilimumab administration in an early-phase clinical trial of relapsed malignancy post alloSCT was not found to induce or exacerbate GVHD.^45^ Nevertheless, special attention should be paid to GVHD when designing clinical studies testing the safety and efficacy of blockade agents of PD-1 or other negative receptors in leukemia post alloSCT. Patients with active GVHD should be excluded from this type of study. If GVHD turns out to be a significant dose-limiting toxicity of checkpoint blockade post alloSCT, other options can be considered to reduce risk, including selecting subsets of T cells that cause no or reduced GVHD^46, 47, 48, 49^ or transferring TCR gene-engineered CD8 T cells.^50^

Our study is limited by small sample size. Given the complexity of the alloSCT procedure, it is challenging to perform studies on large number of samples from groups of patients with comparable conditions other than relapse status. Nevertheless, we were able to analyze samples from 11 patients with AML who received alloSCT, 5 of whom had leukemia relapse and 6 remained in remission at the time of blood collection. The age, gender, status of disease before transplantation, donor stem cell source, conditioning regimen for transplantation and GVHD prophylaxis regimen are largely comparable between the two groups. We detected a statistically significant increase of PD-1^hi^TIM-3^+^ cells in AML patients who had leukemia relapse compared with those who remain in remission. Further collection of patients' samples are underway, the study of which will be helpful to further validate the conclusions. In addition, the majority of patients evaluated in our study received transplantation from filgrastim-mobilized peripheral blood. Whether this phenomenon holds true in two other transplantation settings (bone marrow and umbilical cord blood transplantation) is not known. Although peripheral blood transplantation is currently the most commonly utilized alloSCT, bone marrow transplant or umbilical cord blood transplant is in some clinical scenarios the only or the best option for patients.^51, 52, 53^ It is important to address whether the association between T-cell exhaustion and leukemia relapse is a general feature. If confirmed, targeting T-cell exhaustion as a novel leukemia therapeutic can be widely applied to patients receiving transplantation of variable graft sources.

A key question is whether PD-1^hi^TIM-3^+^ cells are causative of leukemia relapse. We speculate that these cells represent leukemia-specific T cells that are activated initially but become exhausted because of persistent leukemia antigen stimulation. Consistent with our hypothesis, we observed that only relapse patients, not remission patients, have this cell population. To define whether these cells are leukemia specific, major histocompatibility complex tetramers carrying leukemia-specific antigen epitopes would be needed. Although several studies using proteins overexpressed on leukemia cells for vaccination demonstrate promising progress,^54, 55^ leukemia-specific antigens remain undefined in many clinical situations.^56^ Alternatively, antigen specificity can be evaluated by assessing T-cell function after stimulation with the specific antigen-carrying cells; in this case the patient's leukemia cells. Given the exhausted phenotype of these cells, they will unlikely be functional after stimulation, thus making it less possible to detect the antigen specificity. An alternative approach to address this issue is to perform deep TCR sequencing on PD-1^hi^TIM-3^+^ cells.^57, 58^ Antigen-specific T cells have skewed clonal TCRs after antigen stimulation. If PD-1^hi^TIM-3^+^ cells from leukemia relapse patients express limited clonal TCRs, they are likely leukemia-specific T cells. Although beyond the scope of the current study, the archived cells will provide the potential for this type of analysis in an expanded study.

Our finding that PD-1^hi^TIM-3^+^ T cells predict leukemia relapse in AML patients post transplantation has significant clinical impact. Leukemia relapse post transplantation is a devastating disease that carries poor prognosis. Withdrawal of immune suppressors or donor lymphocyte infusions are two means of strengthening GVL by enhancing donor T cell number and activity, and are thus crucial treatment options for these patients. However, they are only effective in early relapse when the leukemia blasts are minimal and when there are still significant donor cells around. Their impact is less in advanced leukemia, in which case induction chemotherapy is necessary to achieve remission. However, intensive chemotherapy is often not tolerable in patients with severe leukemia progression, when multiple complications and comorbidities are present. Thus, early diagnosis of leukemia relapse is the key for successful treatment. Our study identifies PD-1^hi^TIM-3^+^ T cells as a predictive biomarker for leukemia relapse post transplantation, making possible early diagnosis and intervention, thus improving clinical outcome.

In summary, we identified a fraction of T cells, PD-1^hi^TIM-3^+^, that associate and predict leukemia relapse in AML patients post transplantation. Consistent with exhaustion, PD-1^hi^TIM-3^+^ T cells are antigen-experienced cells that are deficient in producing cytokines following stimulation through the TCR. Our study not only provides a predictive biomarker for early diagnosis and facilitating intervention in patients with leukemia relapse, it would also form the basis for developing novel effective leukemia therapeutics by targeting T-cell exhaustion. Agents blocking key mediators involved in T-cell exhaustion are already available and FDA approved for treating several types of solid tumors. Results from our study are important to move this strategy forward to treat patients with leukemia. Leukemia relapse post alloSCT carries an extremely poor clinical prognosis. Results of this clinical study provide pivotal information for an improved strategy to treat this devastating disease.

## Figures and Tables

**Table 1:** Clinical characteristic of patients

*Patient*	*Age*	*Sex*	*Donor*	*Conditioning*	*Disease status*	*Collection time (post alloSCT)*
01	48	M	MUD/PB	Ablative	Remission	6 Months
02	58	M	MUD/BM	Ablative	Remission	6 Months
03	45	M	MUD/PB	Ablative	Remission	5 Months
04	68	F	MSD/PB	Nonablative	Remission	4 Months
05	68	F	MMUD/PB	Ablative	Remission	3 Months
06	67	F	MSD/PB	Nonablative	Remission	3 Months
07	52	M	MSD/PB	Ablative	Relapse	6 Months
08	64	F	MUD/PB	Nonablative	Relapse	5 Months
09	59	M	MSD/PB	Nonablative	Relapse	4 Months
10	53	F	MUD/PB	Ablative	Relapse	3 Months
11	47	M	MSD/PB	Ablative	Relapse	2 Months

Abbreviations: alloSCT, allogeneic stem cell transplantation; F, female; M, male; MMUD, mismatched unrelated donor; MSD, matched sibling donor; MUD, matched unrelated donor; PB, peripheral blood.
