# Supplementary material for: PD-1hiTIM-3+ T cells associate with and predict leukemia relapse in AML patients post allogeneic stem cell transplantation
Source: Blood Cancer J. 2015 Jul 31;5(7):e330–. doi: 10.1038/bcj.2015.58 (PMC4526784; doi:10.1038/bcj.2015.58)
Supplement: Supplementary Figure Legends [file bcj201558x1.doc]

**Supplementary Figure Legends**

**Supplementary Figure 1. Distribution of T_N_, T_CM_, T_EM_, and T_EMRA_ in T cells gated on each fraction of cells based on PD-1 and TIM-3 expression from remission patients.**

(A) Representative dot plots from one remission patient (02). (B) Summary data for five relapse patients (01,02,03,05,06).

**Supplementary Figure 2. T_EMRA_ in AML patients produce more TNF-α, IFN-γ and IL-2 than T_N_, T_CM_, and T_EM_.**

PBMCs were tested by flow cytometry for Production of TNF-α, IFN-γ, and IL-2 upon *in vitro* CD3/28 stimulation. Cytokine release of each subpopulation (T_N_, T_CM_, T_EM_, T_EMRA_) from CD4^+^ (A) or CD8^+^ (B) T cells are shown. Data is representative from one patient with leukemia relapse (patient 11).

**Supplementary Figure 3. Kinetic analysis of PD-1^hi^TIM3^+^ cells in remission patients.**

(A) Representative flow data from one remission patient (02). (B) Shown are kinetic curves of percentage of PD-1^hi^TIM-3^+^ among CD4^+^ or CD8^+^ T cells from four remission patients (01,02,04,06).
