# Supplementary figures and images for: PD-1hiTIM-3+ T cells associate with and predict leukemia relapse in AML patients post allogeneic stem cell transplantation
Source: Blood Cancer J. 2015 Jul 31;5(7):e330–. doi: 10.1038/bcj.2015.58 (PMC4526784; doi:10.1038/bcj.2015.58)

Supplementary Figure 1

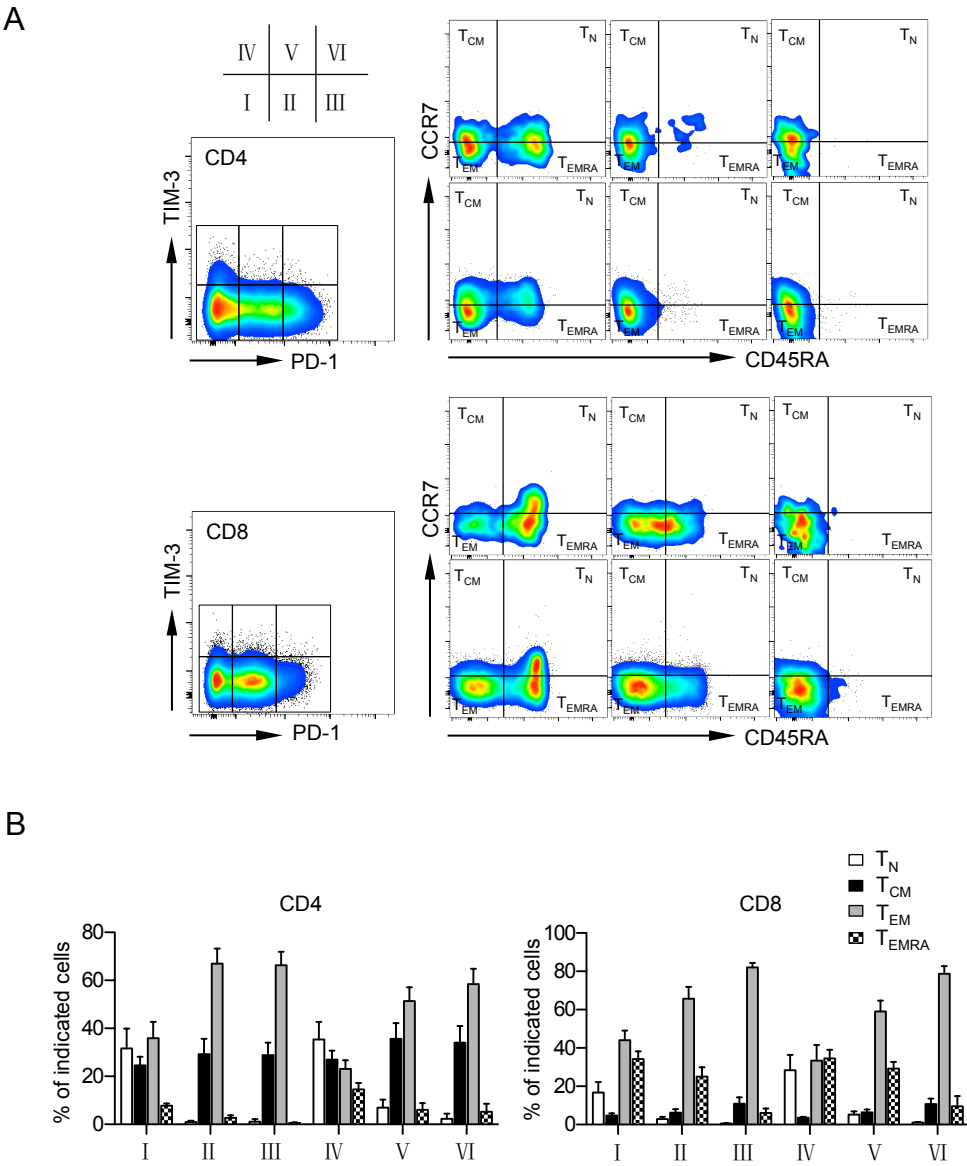

Supplement: Supplementary Figure 1 [file bcj201558x2.pdf]

Supplementary Figure 2

A

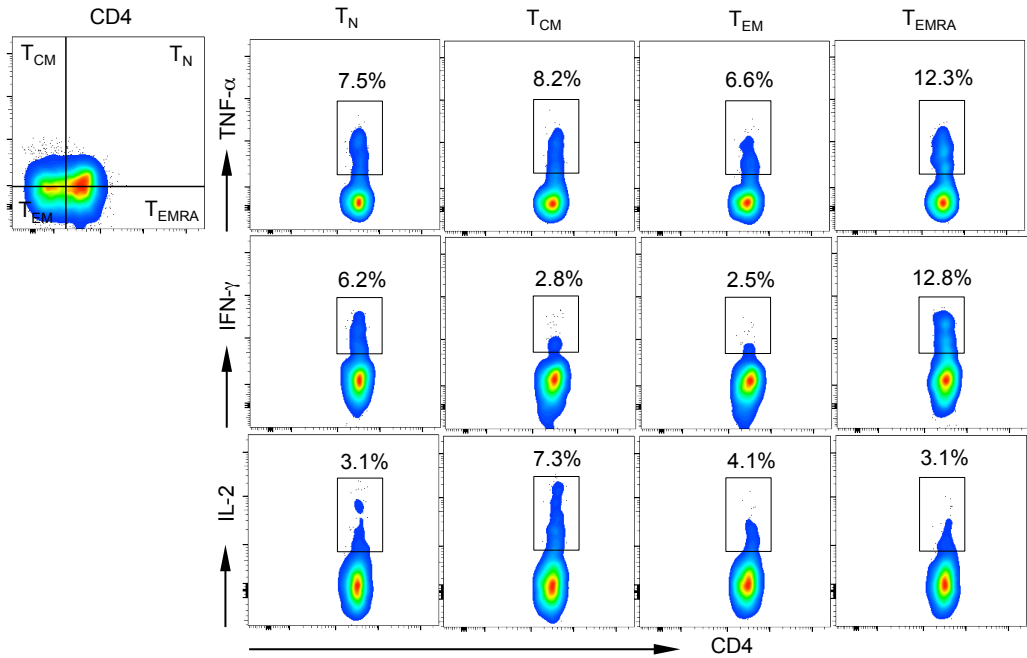

B

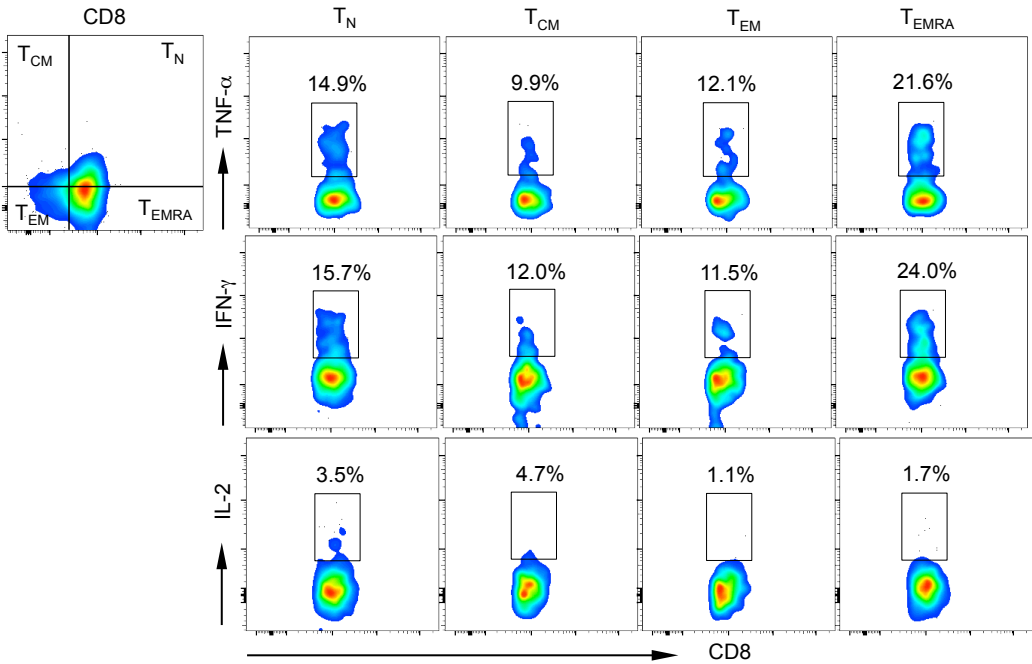

Supplement: Supplementary Figure 2 [file bcj201558x3.pdf]

Supplementary Figure 3

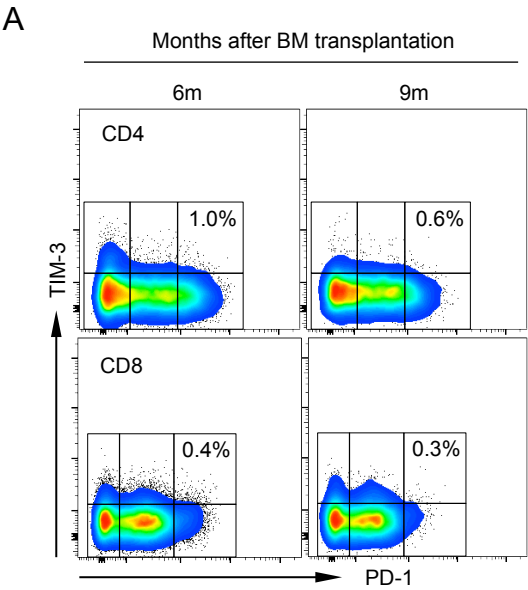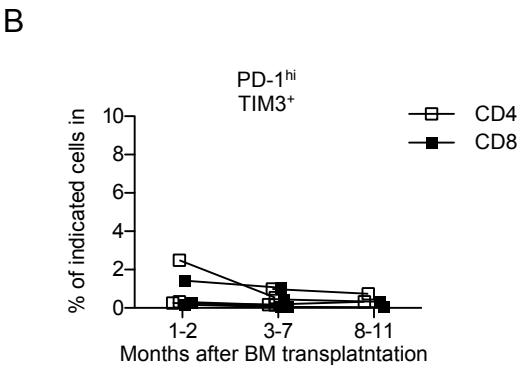

Supplement: Supplementary Figure 3 [file bcj201558x4.pdf]
